# Standardising Culture Medium Safety Testing for Cultivated Meat: Outputs from a Workshop and Case Study

**DOI:** 10.3390/foods15040783

**Published:** 2026-02-21

**Authors:** Ruth E. Wonfor, Kimberly J. Ong, Wei Ng, Jo Anne Shatkin, Reka Tron, Cai Linton

**Affiliations:** 1 Department of Life Sciences, Aberystwyth University, Aberystwyth SY23 3DA, UK; 2 Vireo Advisors, LLC, Boston, MA 02130, USA; kong@vireoadvisors.com (K.J.O.); wei@vireoadvisors.com (W.N.); jashatkin@vireoadvisors.com (J.A.S.); 3 Multus Biotechnology Ltd., 84 Wood Lane, London W12 0BZ, UK; reka.tron@multus.bio (R.T.); cai.linton@multus.bio (C.L.)

**Keywords:** cultivated meat, cellular agriculture, safety testing, food safety, cell culture media, residues, growth factor, method validation, research priorities

## Abstract

Cultivated meat is a novel food and therefore must undergo safety assessments and regulatory review to identify risks and establish appropriate mitigations prior to commercialisation. The culture media used within the cell cultivation process may contain components that lack a long history of use in food, necessitating safety evaluation. However, there is no clearly defined framework outlining the evaluations needed to generate robust and reliable data. The aim of this work was two-fold: first, to convene a multi-stakeholder workshop to identify knowledge gaps related to culture medium safety assessment, and second, to provide a case study addressing one knowledge gap through the evaluation of ELISAs for quantifying growth factors in culture media and cultivated meat products. The workshop findings highlighted critical needs for standardised residue measurement methods, Certificates of Analysis, characterisation of metabolites and breakdown products, as well as open databases. Our case study evaluates the use of ELISAs to quantify six commonly used growth factors for cultivated meat production, comparing their presence in cultivated meat and conventional meat. Growth factor levels varied depending on the medium formulation but were generally reduced to conventional levels or were non-detectable after simulated cooking. Several methodological challenges were identified around the use of ELISAs, such as cross-reactivity between species, limited antibody availability for non-traditional species, and a lack of reference data and standards to support validation of ELISAs and establishment of suitable limits of detection. This work therefore provides actionable guidance for future research in this field for standardisation and emphasises the need for a clearly defined framework and standardised analytical methods to ensure consistent and transparent evaluation of cultivated meat.

## 1. Introduction

Cultivated meat (CM) is a novel food produced from animal-derived cells. Before commercialisation, products must undergo safety assessments and review by food safety regulators to identify risks and appropriate risk mitigation measures. While several CM products have been approved or submitted for approval in multiple jurisdictions, there is currently no harmonised framework for safety testing. This lack of harmonisation has created a strong call for standardisation. Initiatives such as the Cultured Meat and Safety Initiative (CMSI) have begun to assess what is required to advance consistent and transparent evaluation [1].

One of the key safety considerations involves the assessment of culture media, which supply nutrients, growth factors, and other molecules to support cell growth and differentiation [1,2,3,4,5]. Medium formulations contain a wide array of common food components, such as amino acids and salts, but also some that may lack a long history of use as food additives. Traditional cell culture has relied on foetal bovine serum (FBS), yet for CM to be viable, serum-free medium formulations are being developed. These serum-free formulations may include recombinant proteins, such as growth factors. Although the medium is removed during cell harvest, medium residues may remain in the final cell biomass, potentially at higher concentrations than those found in conventional meat [5].

There are currently gaps in both publicly available data and methods for assessment of culture media and subsequent medium residues in the final product. Suppliers often lack clarity on the content of Certificates of Analysis (CoAs) for CM-specific ingredients, and there are no agreed standards for safety-relevant specifications. Publicly available information on the composition, stability, or behaviour of many medium components is sparse, limiting the ability to benchmark or compare across products. At the same time, there is no consensus on the most appropriate analytical methods for detecting or quantifying residues, metabolites, or degradation products or their bioactivity in complex food matrices. These gaps make it difficult for companies to develop regulatory dossiers that are comprehensive and aligned with reviewer expectations, creating inefficiencies across the sector.

As discussed in refs. [1,6], growth factors are among the more significant components requiring methods for assessment. These bioactive proteins are essential to support cell viability and proliferation, but as biologically active proteins, their presence in food products warrants careful characterization [2,4,5,7]. Growth factors are produced by animal cells and therefore will be present in conventional meat, although quantification methods and reference levels for conventional meat are lacking. While residues of growth factors may be present in final CM products, exposure will depend on their stability and loss of bioactivity during cooking and digestion. However, there is currently no comparative data on residues in CM relative to conventional meat [7].

As shown in refs. [1,5], regulators and risk assessors require robust, validated analytical methods to detect, quantify, and characterise exposure risk of such components in final food products. Currently, no standardised analytical approach exists for growth factor quantification. These methods underpin risk assessment, help establish limits of detection (LODs), and support comparisons with conventional foods. At present, gaps in analytical methodologies, particularly for complex protein analytes, pose a barrier to consistent safety demonstration across the CM sector.

Recognising these gaps, the CMSI convened a workshop to discuss needs and identify research priorities for analytical methods in culture medium safety assessment. Diverse stakeholders from industry, academia, government, and non-profit organisations identified research priorities and charted a path toward standardised testing and data sharing. This was critical for identifying key requirements for new analytical methods or an entire industry.

In addition to summarising this workshop, this paper also presents a case study to develop ELISA-based methods for growth factor detection, illustrating technical challenges in one of the workshop’s priority areas. By integrating the consensus priorities with specific method development experience, this paper proposes a roadmap for advancing culture medium safety assessment in CM, a novel approach for analytical method development.

## 2. Workshop Overview

This workshop built upon CMSI workshops in 2024 and 2025 that had identified analytical test methods and standards for culture media as a priority focus area. The CMSI workshop on culture medium safety was held on 25 June 2025, at Imperial College London, UK. The session was designed to identify and refine research priorities for safety evaluations of culture medium inputs, to develop actionable research roadmaps for analytical method development, standards, and data infrastructure, and to foster cross-sector collaboration among producers, regulators, and analytical experts. The event was conducted under the Chatham House Rule to promote candid discussion.

A total of 41 participants attended, representing academia (29%), industry (61%), government (7%), and non-profit organisations (2%). The organising committee included representatives from Aberystwyth University, acib (Graz, Austria), Hoxton Farms (London, UK), Imperial College Bezos Centre, Ivy Farms (London, UK), Qkine (Cambridge, UK), RSSL (Reading, UK), Vireo Advisors (Boston, MA, USA), and Multus (London, UK), with support from New Harvest (Amsterdam, The Netherlands). The full agenda is provided in Supplementary Materials S1.

The workshop participants identified five major focus areas for advancing analytical capabilities and standards for culture medium safety in CM: residue measurement methods, developing standardised Certificates of Analysis, characterisation of metabolites, evaluating mixtures, and development of open databases. These outcomes reflect the sector’s recognition that coordinated, collaborative research is needed to reduce duplication, align with regulator expectations, and streamline safety assessments.

### 2.1. Residue Measurement Methods

Focus areas included prioritising inputs without a history of safe use or with potential hazards, defining appropriate LODs, and selecting and validating analytical methods such as ELISA and mass spectrometry (MS). Discussions emphasised that regulators expect companies to provide data and information on potential residues in the final product, including toxicological data and quantification demonstrating residue levels are safe for consumption. Proposed research involved generating baseline data for residues in conventional meat to serve as comparators, developing and validating methods via interlaboratory studies to ensure reproducibility and build confidence with regulators, creating centralised residue data repositories, and establishing risk-based testing strategies for priority inputs. Discussions considered the use of non-targeted methods (e.g., MS) to screen medium formulations and identify priority residues for subsequent testing using targeted methods (e.g., ELISA). Challenges included unknown medium compositions, intellectual property barriers, limited method availability, and the cost of advanced instrumentation. The potential impact would be enabling targeted, regulator-justified testing strategies and strengthening consumer trust.

### 2.2. Certificates of Analysis (CoAs)

Focus areas included developing standardised CoAs for CM-specific inputs, defining necessary quality and safety checks. Discussions identified potential parameters relevant to CoAs (e.g., identity, purity, potential contaminants, physical and chemical properties, stability, functionality, allergenicity, sustainability) and assessed the current landscape of standard testing methods for these parameters, categorising them by their availability and applicability to culture media. Proposed research included assessing bioactivity, degradation products, allergenicity, and recombinant DNA content in culture medium components; developing and validating methods for input assessment; and creating a CoA template suitable for CM. Challenges included protecting proprietary formulations, accrediting methods, and addressing batch variability. The potential impact would be the acceleration of regulatory approval, reductions in production failures, and increases in supply chain resilience.

### 2.3. Metabolites and Breakdown Products

Focus areas included characterising metabolites from CM production and identifying compounds of potential safety concern. Proposed research involved using metabolomics to compare metabolites and breakdown products in CM and conventional meat and developing standardised test methods and acceptance criteria for metabolites of concern. Participants also discussed generating metabolomics data for multiple industrially relevant cell lines, media, and process conditions as well as exploring the use of these data to develop an in silico tool to predict the formation of metabolites of potential concern in new medium formulations or manufacturing conditions. Challenges included complex data analysis, variability across species and processes, a lack of reference datasets, and the need for standard safety assessment approaches for metabolites and breakdown products. The potential impact would be the advancement of streamlined medium development and targeted safety evaluation.

### 2.4. Mixtures (Lysates and Hydrolysates)

Focus areas included the development of methods for identifying and quantifying known and unknown components in complex mixtures, focusing on extracts (e.g., plant hydrolysates). Participants proposed using a model system, such as hydrolysate from yeast or soy, to develop an analytical approach for characterising complex mixtures. Proposed research involved the use of analytical methods to fractionate the complex mixture by size and physiochemical properties and enable the identification and quantification of known components. Unknown components could be further characterised through analysis of chemical properties, structure, and functional groups to assist in identification, followed by allergenicity and toxicity testing to support safety evaluation. Challenges included variability across batches and suppliers as well as the need for validated and accredited methods. The potential impact would be a clear safety evaluation pathway for complex mixtures in culture media.

### 2.5. Open Databases

The development of centralised, publicly accessible databases for the growth factors and antibodies used in CM production and analytics was highlighted as critically important. Proposed research included collecting and sharing key data on growth factors (e.g., species origin, stability, production organism) and antibodies (e.g., specificity, clonality, production organism) in open databases. Participants also discussed developing shared protocols and test methods, including for growth factor quantification and antibody validation. Challenges included intellectual property and confidentiality concerns, the high cost of data development, the need for ongoing database maintenance and the lack of existing CM-specific repositories. The potential impact would be reduced duplication, lower costs, faster time-to-market, and stronger regulatory submissions.

## 3. Case Study: Initial Method Development for Growth Factor Detection

The case study aimed to develop sensitive, reproducible ELISA-based methods for quantifying select growth factors in CM production. This work addressed one of the workshop’s highest-ranked priorities: residue measurement methods. The project measured growth factor levels in three medium types (FBS and two Multus serum replacer formulations) and assessed resulting growth factor residues in CM produced from each medium formulation, in both non-heated (raw) and heated (cooked) CM. The CM was also compared to conventional meat as a reference control.

### 3.1. Materials and Methods

#### 3.1.1. Media

All three media (FBS and two Multus serum-free formulations) were all added at a 10% volume with a basal medium of DMEM/F-12 with L-glutamine, HEPES and phenol red (Gibco, Thermo Fisher Scientific, Waltham, MA, USA). The two serum-free formulations from Multus (Multus Biotechnology, London, UK) both comprised the same components, but medium 1 utilised a formulation with lower concentrations of several components compared to medium 2 (Table 1). All components added to the Multus medium formulations were recombinant human protein sources. For comparison, a standard cell culture medium was prepared with 10% FBS (Gibco, Thermo Fisher Scientific). The DMEM/F-12 with 10% of each of the three medium types will subsequently be referred to as SMA (Multus medium 1), SMB (Multus medium 2) and FBS (FBS).

#### 3.1.2. Cultivated Meat and Conventional Meat Collection

CM samples were prepared using ovine myosatellite cells (Extracellular Ltd., Bristol, UK). Cells were cultured in the three different medium types, FBS, SMA and SMB (as described in Section 3.1.1), within tissue culture vessels (Thermo Fisher Scientific) at 37 °C, 5% CO_2_. Three batches of cells were produced for each medium type. Harvested cells and medium samples were stored at −80 °C until subsequent analysis.

Conventional lamb meat was used as a control and collected from three animals slaughtered as a part of standard commercial meat operations from a local abattoir. Samples were collected within 30 min of slaughter, immediately snap frozen on dry ice and stored at −80 °C until subsequent analysis.

#### 3.1.3. Heating Samples to Emulate Cooking

Media and meat (cultivated and conventional) were heated to emulate cooking, following UK guidance for lamb meat reaching 70 °C for 2 min [8]. Samples were placed on a prewarmed heat block and allowed to reach 70 °C for 2 min. A control tube was used to verify that the internal mass or liquid volume in the tubes reached and maintained 70 °C for the full 2 min. Following heating, samples were stored at −80 °C until subsequent analysis.

#### 3.1.4. Protein Extraction and Quantification from Cultivated Meat and Conventional Meat

All CM and lamb meat samples, heated and non-heated, were homogenised on ice using a Dounce homogeniser and cell extraction buffer (Invitrogen, Thermo Fisher Scientific, Waltham, MA, USA) at a ratio of 1 g (wet mass):8 mL buffer, following the manufacturer’s instructions. Extracted samples were aliquoted and stored at −80 °C. All media and extracted meat samples (non-heated and heated) were assessed for protein content using the Bradford assay [9].

#### 3.1.5. Enzyme-Linked Immunosorbent Assays (ELISAs)

Commercially available ELISAs were purchased for 6 growth factors: EGF, FGF-2, HGF, IGF-1, PDGF and TGF β1 (sources and ranges in Table S1). For each growth factor, human-, bovine- and ovine-specific kits were used to detect growth factors originating from the SMA and SMB media (human) and FBS media (bovine) as well as CM and conventional meat (ovine). All ELISAs were run as per the manufacturer’s instructions. Dilution tests were completed for each sample type to ensure samples fell within the standard curve range. Following establishment of correct dilutions, all samples were run on one plate for each protein and species. Intra-assay CVs were below 10%, except bovine FGF-2 (10.64%).

All media and meat samples were tested for every target growth factor. However, samples were only analysed on the species-specific ELISA relevant to the sample type. For example, SMA and SMB media containing recombinant human growth factors were run on the human-specific ELISAs, and CM produced with SMA and SMB media were run on both the human (to test for medium residue)- and ovine (to test for natural presence produced by the cells)-specific ELISAs. Due to the high sequence similarity between species, cross-reactivity was also assessed for each growth factor target. Therefore, control samples were run on ELISAs where the species target should not be present in the sample (e.g., SMA samples only containing human proteins were tested on bovine and ovine ELISA plates). Control samples were not run with full batch replication but just with one non-heated batch. Each plate included a standard set of controls: a positive control (medium with standard spiked), a negative control (medium alone), a cell extraction buffer control and a blank. ELISAs were read on a FLUOstar Omega Microplate reader (BMG Labtech, Ortenberg, Germany), at absorbencies as per the manufacturer’s instructions.

All ELISA optical density (OD) readings were quantified against the standard curve for each plate. Samples were multiplied by the dilution factor to determine the absolute protein concentration. Growth factor concentrations in cultivated and lamb meat samples were converted to a mass per mg of solid sample (wet mass), using the extraction wet-mass-to-buffer ratio.

#### 3.1.6. Statistical Analysis

All statistical analysis was completed in Genstat (Version 22, VSN International Ltd., Hemel Hempstead, UK) and results are reported as mean ± standard error of the mean (SEM). Prior to statistical analysis, a mean was taken from technical duplicates and data were tested for normal distribution. Statistical significance was set at *p* < 0.05 and Tukey’s post hoc test was utilised where significant differences were demonstrated after ANOVA.

Protein content following Bradford quantification was assessed within medium and meat samples. Medium samples were analysed via a one-way ANOVA to assess the effect of heating on protein content of the media. Meat samples (cultivated and lamb) were analysed via two-way ANOVA to assess the effect of heating on protein content and differences in protein content between the meat types.

SMA and SMB media and CM produced from these media were assessed via one-way ANOVA to assess the effect of heating on human protein concentration. FBS media and the corresponding CM samples were assessed with a paired *t*-test to assess the effect of heating on bovine protein concentration. Ovine cultivated and lamb meat samples were assessed using a two-way ANOVA to evaluate the effect of heating treatment on ovine protein concentration and the difference in protein content between CM and lamb meat. In cases where concentrations of ovine growth factors were below the limit of detection for all heated samples, a one-way ANOVA was used to compare meat types only.

### 3.2. Results

#### 3.2.1. Protein Content of Media and Meat Samples

**Figure 1:**
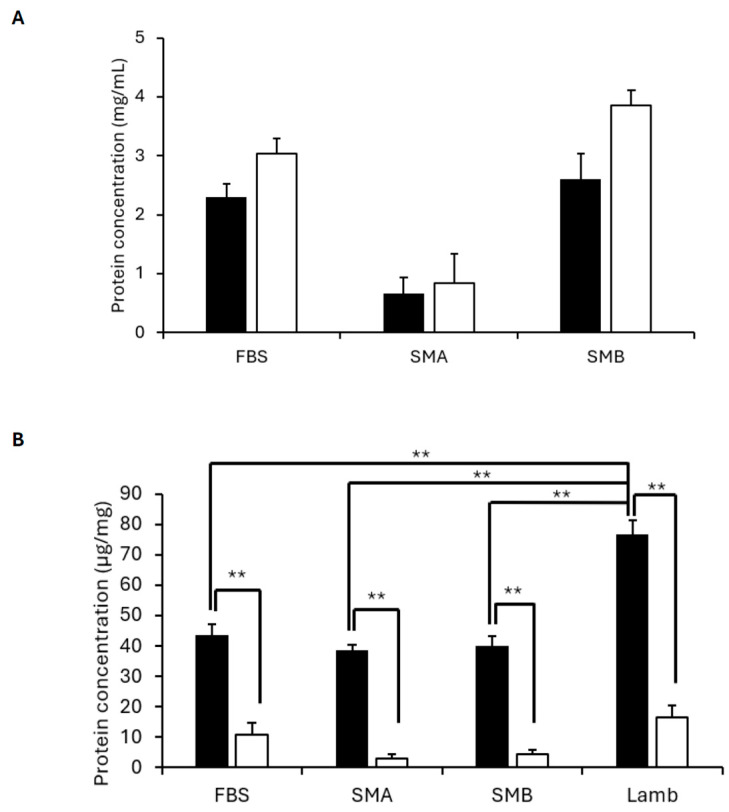
Protein content as quantified by Bradford assay in non-heated (closed bars) and heated (open bars) samples: medium types (**A**) and cultivated meat and lamb meat, following protein extraction (**B**). Data are expressed as means, with error bars demonstrating the SEM. ** demonstrates *p* < 0.01.

Protein quantification via a Bradford assay of non-heated and heated media demonstrated no effect of heating on the overall protein content of the media (*p* > 0.05; Figure 1A). However, heating significantly reduced the protein content of all CM types and lamb meat (*p* < 0.01; Figure 1B). Furthermore, when assessing differences in protein content between meat types, it was demonstrated that there was significantly more protein in lamb meat compared to CM on a standardised wet mass (*p* < 0.01; Figure 1B).

#### 3.2.2. Growth Factor Quantification in Media, Cultivated and Conventional Meat

##### Culture Media

**Figure 2:**
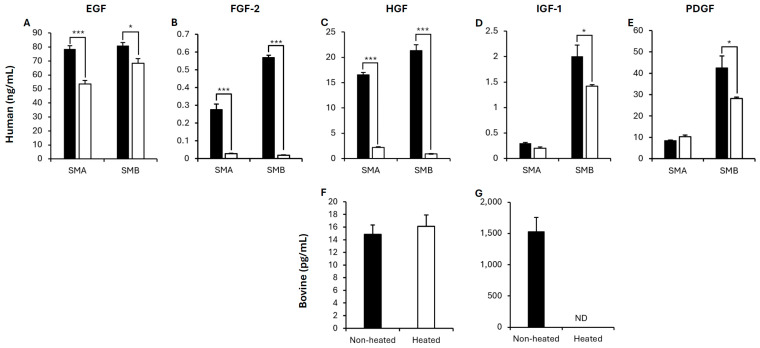
Growth factor quantification in SMA, SMB (**A**–**E**) and FBS (**F**,**G**) media on human (**A**–**E**) and bovine (**F**,**G**) ELISAs, when media were non-heated (closed bars) and heated (open bars). Data are expressed as means with error bars demonstrating the SEM. *** and * demonstrate *p* < 0.001 and *p* < 0.05, respectively. ND demonstrates samples that were not detectable below the ELISA range. There was no detection of EGF, FGF-2 and PDGF in FBS media on the bovine ELISA.

Growth factor concentrations were quantified in non-heated and heated SMA and SMB media using human ELISAs. hTGF β1 was the only growth factor not detected. All other growth factors (hEGF, hFGF-2, hHGF, hIGF-1 and hPDGF) were detected (Figure 2A–E). Heating significantly reduced the concentration of growth factors compared to the non-heated media in all cases (*p* < 0.05; Figure 2A–E), except for hIGF-1 and hPDGF in SMA media (*p* > 0.05; Figure 2D,E).

Bovine ELISAs were used to quantify growth factor concentrations in non-heated and heated FBS media. Only bHGF and bIGF-1 were detected in non-heated FBS media above the lowest limit of quantitation (LLOQ) (Figure 2F,G). Heating had no effect on bHGF concentration in FBS media, compared to non-heated media (*p* > 0.05; Figure 2F), but bIGF-1 was reduced to undetectable levels following heating, demonstrating a reduction in concentration compared to the control (Figure 2G).

##### Cultivated and Conventional Meat

**Figure 3:**
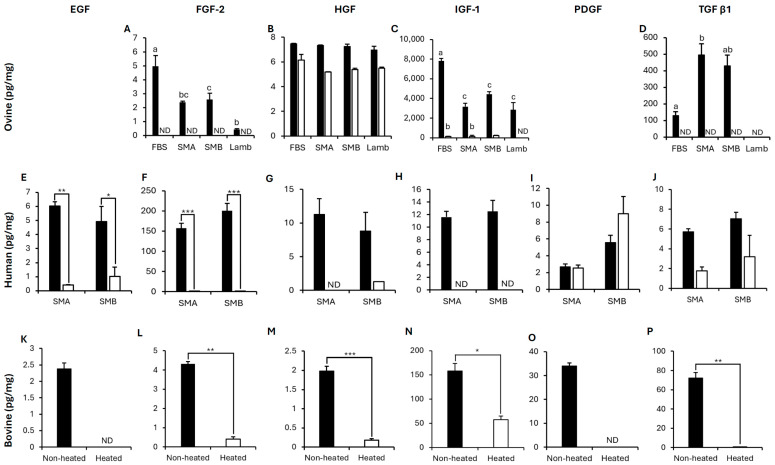
Growth factor quantification in ovine cultivated meat produced with FBS, SMA or SMB media and conventional lamb meat. All cultivated meat and conventional lamb meat were quantified via ovine ELISAs to test for growth factors produced by the cells (**A**–**D**). Both SMA and SMB produced cultivated meat were quantified via human ELISAs (**E**–**J**) and FBS produced cultivated meat via bovine ELISAs (**K**–**P**) to test for medium residues. Closed bars demonstrate non-heated and open bars demonstrate heated meat. Data are expressed as means with error bars demonstrating the SEM. ***, ** and * demonstrate *p* < 0.001, *p* < 0.01 and *p* < 0.05, respectively. Bars not sharing a letter are significantly different (*p* < 0.05). ND demonstrates samples that were not detectable below the ELISA range. In SMB heated, oIGF-1 and oHGF were only quantified in one batch and therefore error bars are not presented, and the sample type was removed from the statistical analysis.

To assess growth factors in CM produced with FBS, SMA and SMB, ovine ELISAs were used to quantify the growth factors potentially produced by the cells and to compare them to levels naturally found in lamb meat. oEGF and oPDGF were undetectable in all sample types. oFGF-2, oHGF and oIGF-1 were detected in non-heated CM and conventional lamb (Figure 3A–C), whereas TGF β1 was only detected in non-heated CM (Figure 3D). There was no difference in the concentration of oHGF between meat types (*p* > 0.05; Figure 3B). However, there were differences in oFGF-2, oIGF-1 and oTGF β1 between meat types. Concentrations of oFGF-2 and oIGF-1 were significantly greater in FBS-produced CM compared to SMA- and SMB-produced CM and conventional lamb (*p* < 0.05; Figure 3A,C). oFGF-2 and oIGF-1 concentrations in SMA- and SMB-produced CM were not significantly different to conventional lamb, except for SMB-produced CM, which demonstrated a significantly greater concentration of oFGF-2 compared to conventional meat (*p* < 0.05; Figure 3A). oTGF β1 was significantly greater in SMA-produced CM compared to FBS-produced CM (*p* < 0.05; Figure 3D).

For oFGF-2, oIGF-1 and oTGF β1, heating the cultivated and conventional meat either significantly reduced the concentration of the growth factor (oIGF-1 FBS and SMA: *p* < 0.001; Figure 3C) or reduced it to undetectable levels (oFGF-2 (Figure 3A), oIGF-1 (SMB and Lamb (Figure 3C), oTGF β1 (Figure 3D)). Across all meat types, heating resulted in a significant reduction in oHGF compared to the non-heated samples (*p* < 0.001). However, there was no significant interaction between the different meat types and heating (*p* > 0.05; Figure 3B).

To assess whether medium-derived growth factors (human in SMA and SMB, bovine in FBS) could be detected in CM, SMA- and SMB-produced CM were tested on human ELISAs, and FBS-produced CM on bovine ELISAs. All growth factors were detected in non-heated CM in both human (Figure 3E–J) and bovine (Figure 3K–P) ELISAs. Heating the CM either significantly reduced the concentration of the growth factor in the CM (*p* < 0.05; Figure 3E,F,L–N,P) or reduced it to undetectable levels below the LLOQ (Figure 3G,H,K,O), except hPDGF (*p* > 0.05; Figure 3I) and hTGF β1 (*p* > 0.05; Figure 3J).

Growth factor concentrations quantified in the CM varied between the different species targets. For example, ovine and bovine FGF-2 remained <6 pg/mg across all meat types (Figure 3A,L), whereas human FGF-2 reached a mean of 157 and 200 pg/mg in SMA and SMB non-heated CM, respectively (Figure 3F).

To test for potential species cross-reactivity, non-relevant samples were run on each ELISA. For example, technical replicates of SMA and SMB media (human growth factors) were quantified on the bovine and ovine ELISAs. Similarly, a technical replicate of lamb meat samples was quantified on both the human and bovine ELISAs. As expected, the species-specific antibodies demonstrated a high level of cross-reactivity, with detection of non-target species in at least one sample type (media or meat) for every growth factor (Table S2).

### 3.3. Key Findings

This case study provides the first evaluation of a suggested and common method to quantify medium residues and native growth factors in CM products. Furthermore, we report the first measurements of growth factors commonly used in CM culture media both within the CM product and conventional meat before and after cooking. This work provides valuable information for the CM industry and establishes a baseline to stimulate discussions among researchers and at the CMSI workshop, described here.

Analysis of lamb CM demonstrated that all growth factors, except oEGF and oPDGF, could be detected by ELISA. Interestingly, neither oEGF nor oPDGF was quantifiable in lamb meat. Growth factor concentrations varied between CM produced with different media and compared to lamb meat, suggesting that medium composition influences CM growth factor content. This was evident in the case for IGF-1, where the concentration of oIGF-1 was significantly greater in the CM produced by FBS media, aligned with the much higher concentration quantified in FBS media relative to the Multus media.

There is evidence that some media may drive endogenous production of growth factors due to the presence of other components in the media. The content of oFGF-2 in FBS-produced CM was higher compared to the Multus media-produced CM, yet on analysis of the FBS and Multus media, bFGF-2 was undetectable and hFGF-2 was detectable, despite similarities in the ranges of the ELISAs. Therefore, there is potentially a driver in FBS that stimulates further production of oFGF-2 by the myosatellite cells. FBS contains a multitude of undefined bioactive factors [10], unlike the methodically formulated Multus media. For example, derivatives of Vitamin A are demonstrated to increase FGF-2 production in endothelial cells [11] and are well known to be present in numerous biological matrices, including FBS [10,12].

Interestingly, the concentrations of oFGF-2, oHGF and oIGF-1 in CM produced from Multus media, especially the lower-concentration formulation (SMA), were not significantly different from lamb meat, although this was not the case for oTGF β1. Yet FBS-produced CM had significantly different concentrations of oFGF-2, oIGF-1 and oTGF β1 compared to lamb meat. These findings support a use for carefully defined medium formulations in the CM industry, where the presence of other factors which can potentially promote the unnatural presence of components of safety concern, such as growth factors, can be minimised. Although plant hydrolysates and other mixed-media formulations are a popular area of research because they are readily derived from food sources and can reduce dependence on costly chemically defined components [13,14,15,16], their use still raises safety concerns due to the complexity of these mixtures, similar to FBS. As such, it is imperative to quantify the presence of components such as growth factors to assess the impact on cell behaviour during production and to detect and evaluate residual medium components in the final CM product. This need for systematic characterisation was highlighted as a key research theme in the CMSI workshop.

The case study also determined the effect of cooking on growth factor content in CM and the media themselves. Growth factors are often thermally unstable and several, such as IGF-1 and EGF, have been demonstrated to reduce in bovine and human milk following heating at similar temperatures to those used in the present study [17,18]. It was therefore hypothesised that cooking was likely to reduce growth factor presence in CM and conventional meat. In the majority of cases, the study did find that the concentration of growth factors was significantly reduced to a low concentration or to undetectable levels in both the meat (CM or conventional) and media. Interestingly, CM growth factor content was usually reduced to levels similar to that of the conventional meat or to levels below the LOD, which is promising for human consumption of CM products if growth factors are greater in raw CM compared to conventional meat. These findings are consistent with the well-established denaturing of many meat proteins during heating [19] and supported by the quantification of the protein in the present study. However, it must be noted that some meats are consumed raw. Therefore, safety assessments must consider the risks of raw or undercooked CM beyond the standard food undercooking risks, such as food poisoning. Furthermore, with the development of more thermostable growth factors to be used in CM production [20], consideration will need to be given to the assessment of the behaviour of their residues in CM products and not rely solely on data from growth factors produced with native sequences and conventional methods. For example, an engineered FGF-2 demonstrated an in vitro functional half-life at 37 °C of more than 20 days compared to 10 h for the wild type [21], which will likely impact the persistence of residues in the final product.

It must be noted that for this work we stored both the CM and conventional meat at −80 °C immediately after harvesting to preserve growth factors for analysis. In contrast, conventional lamb carcasses would usually be hung for several days prior to sale to improve eating quality [22]. Subsequently, meat follows a standard supply chain of both transport and shelf storage prior to sale that both conventional meat and CM would be subject to. Even if meat is stored at 4 °C, it is likely that transport, storage and shelf-life of both conventional and CM post-harvest would still result in a natural reduction in growth factor concentration within the meat due to their thermal instability and short half-lives. Furthermore, the immediate harvesting and freezing of CM in the present study does not reflect practices in industry which are likely to include a number of post-harvest processing steps, such as washing of the cell biomass prior to product preparation [23,24,25,26,27]. Such post-harvest processing activities are likely to reduce the content of residual biological components, such as growth factors. As such, once reliable analytical methodologies for quantifying growth factors and other medium residues are validated, studies should be completed in real-world situations to quantify residues in the product post harvesting and through the final production processes. It would also be beneficial to include quantification of any remaining residues alongside shelf-life studies to better understand the product that the consumer is likely to purchase.

As for many components of growth media, including growth factors, quantification data in farmed animals are available for serum, plasma or milk, but not meat itself [7], creating a gap in reference values to determine ‘safe’ or typical exposure levels. While milk data can provide a basis for understanding current exposures, a more comparable reference for CM is conventional meat. Such a reference matrix is likely to be whole meat, although it is acknowledged that whole meat tissue contains a number of different tissue types, rather than myosatellite cells alone, as used in the CM in the present case study. Nevertheless, empirical data for meat and the individual cells that make up the whole tissue is still required. Our case study provides the first direct quantification of a range of growth factors used in CM production in conventional meat. Huang et al. [7] estimated levels of growth factors in cultivated cells and mammalian tissue; when compared to our findings, all but oIGF-1 fall below their estimated values for CM and the measured values for mammal tissue. Nevertheless, broader datasets across variables such as species, age of animal and meat processing methods are needed to allow the wider industry and regulators to provide data-driven knowledge of the current exposure levels in food. Future work should also incorporate testing for a number of different livestock species that are commonly used in cultivated meat production, such as bovine and porcine origin cells. Further complexity arises from the use of heterologous growth factors (e.g., bovine growth factors used to cultivate ovine cells). While recombinant growth factors often share the same amino acid sequence as their native counterparts, cross-species sequence homology differences can complicate detection, antibody specificity, and interpretation of bioactivity measurements [7]. This study utilised growth factors from different species, either recombinant human (Multus media) or wild-type bovine (FBS), to produce ovine CM. Therefore, ELISAs designed for different species were used to assess endogenous ovine growth factors and potential human or bovine growth factor medium residues. All tested growth factors could be detected via the human and bovine ELISAs in non-heated CM. However, with the exception of EGF, the growth factors assessed are relatively well conserved between human, bovine and porcine protein sequences [7]. Although ovine sequence homologies have not previously been characterised, similar homology is assumed. Consequently, cross-reactivity between species-relevant antibodies in the ELISAs was highly likely, indicating that detection on the human and bovine plates was unlikely to represent medium residues alone. This interpretation was supported by control samples analysed using non-relevant species ELISAs, which demonstrated detection of all growth factors in at least one non-relevant assay, including EGF, for which the bovine sequence is not well conserved [7]. Furthermore, growth factor concentrations varied inconsistently across species-specific ELISAs, suggesting that the antibodies were unable to efficiently detect non-target species. Collectively, these findings highlight the challenges associated with quantifying growth factors in CM when culture media contain growth factors derived from heterologous species. Therefore, it is more practical to better understand acceptable levels of each growth factor and then measure total growth factor content in the final CM product, with the understanding that the measured value represents both medium residues and native growth factors. Ideally, where several species sources are used for a specific target, analytical tests should be validated to ensure that they can accurately detect the species sequences that will be found in the native CM product, as well as the medium growth factor species sequences. Furthermore, if the ELISAs in the present study were truly detecting residues from media, it would be expected that they would be detected at a lower concentration compared to that determined via the ovine ELISAs (in theory quantifying native growth factors). Yet this was not always the case; for example, FGF-2 was detected at a much higher concentration for hFGF-2 compared to oFGF-2 in SMA- and SMB-produced CM. These results therefore further highlight potential issues of sensitivity and specificity of the ELISA antibodies used in the present study.

As the study used commercially available ELISAs, we did not aim to validate any commercial kits, and kits were used as per the manufacturer protocols. ELISAs are a commonly used method for research and clinical use but are known to have several analytical and technical flaws [28,29]. Moreover, they can be costly, and it becomes increasingly difficult and more expensive to source commercially available kits for ‘non-traditional’ species that are not of common research interest, such as human or mouse targets. Protein engineering (e.g., for more stable growth factors) further complicates detection; this may alter protein sequences and structures which render the antibodies raised against the wild type to be ineffective or have less affinity. These challenges suggest that many companies will use in-house-developed ELISAs for cost effectiveness and to be able to utilise antibodies optimised for the relevant species. The present case study highlights the importance of ensuring that ELISAs are properly validated and repeatable. Future work in this area should assess the sensitivity and specificity of ELISAs used and provide guidelines on quality control, as well as assessment of interlaboratory variability if the tests are being used across companies or laboratories. Antibodies should be validated against relevant isoforms for each target to ensure appropriate affinity for all relevant protein structures. As such, well-characterised, species-relevant standards will be required for the provision of quality control within the validation process, although it is difficult to provide reference material that models the real food matrix [29].

Quantification of growth factors was further influenced by assay sensitivity and the suitability of sample protein extraction. The species-specific ELISAs used in this case study often differed substantially in ranges and LOD, with the bovine and ovine plates having higher LODs and therefore reduced ability to detect lower concentrations. There were several samples that could not be quantified, namely all meat samples for oEGF and oPDGF and several heated meat and medium samples. Determining appropriate LODs or LLOQs requires a balance between suitable sensitivity at a low concentration and the cost of the test. Without established reference values in conventional meat or clear information on what constitutes a ‘safe’ dietary exposure, it is difficult to define suitable LODs and LLOQs for safety assessments. As such, further research is required to provide empirical data to underpin guidance on LODs/LLOQs required for validated ELISAs. Additionally, to ensure accurate quantification within the LOD/LLOQ, sample protein extraction must be considered to prevent false negatives. The buffer used must be appropriate for the analytical test being completed. We utilised a commercially available extraction buffer after having initially tested in-house-prepared standard buffers that either interacted with the ELISAs or did not effectively extract or stabilise the growth factors during the extraction process so that no growth factor was detected.

This study focused on one group of medium components, growth factors. Six different targets known to be included in serum-free medium formulations were tested; however, no other proteins or components were evaluated. Therefore, this body of work reflects only a small portion of the residues which would need to be tested [23,24,25,26,27]. Large-scale and continued use of ELISAs poses a substantial analytical and financial burden. Therefore, for this reason and due to the methodological limitations listed previously, future research on alternative analytical methods is needed. Multiplex immunoassays would be suitable but likely need to be developed for the range of targets specific to CM. The method with the most promise is likely to be mass spectrometry (MS), which would allow multiple targets to be quantified at one time within each sample, providing a high-throughput alternative. Although expensive, it is likely to be cheaper and require a lower sample mass than completing multiple ELISAs per sample for a large range of targets, although access to expensive MS equipment is a hinderance and will result in validated analytical labs being used for the required assessments. Furthermore, using non-targeted sequencing would be useful for more complex medium formulations (e.g., hydrolysates). However, at present, methodological development is required. Huang et al. [7] completed a literature review to assess currently measured levels of several growth factors in studies related to food safety and oral exposure. The majority of analytical methods used were ELISAs or RIAs, with none using MS, further highlighting a need for methodological establishment. In preliminary trials, we tested detection of the growth factors of interest in this study in the media, CM and lamb meat. A range of proteins were detectable in all samples, but detection of the growth factors was not possible in our initial methods, using both a GeLC and gel-free approach with LC-MS/MS. These results were utilised to initially screen methods for this study and so are not the focus of this publication, due to their preliminary nature. However, they did provide us with a starting point for a framework for further research required in this area. Key areas of focus will need to include stabilisation of proteins with short half-lives (e.g., growth factors) in the sample preparation process; fractionation of samples into protein sizes to prevent the overloading of large and abundant proteins, such as albumin, which mask detection of the smaller and less abundant proteins; and targeted sequencing methodologies, as well as additional cell types.

### 3.4. Key Challenges

The present case study allowed us to compile a list of key challenges for the assessment of growth factors in relation to culture medium safety testing:

Sample preparation and extraction

- Sample processing or storage after extraction: presence in CM will likely differ between products stored for quantification immediately after harvest and those following a usual processing and supply chain storage pipeline.
- Buffer selection and the extraction process influence results, dependent on the analytical method used.

ELISA

- Cross-reactivity between antibodies across species is an issue when species-specific growth factors are not used.
- Variability in kit sensitivity, specificity, LODs and LLOQs.
- A need for validation and QC guidance for both commercially available and in-house-developed ELISAs to ensure that produced results are comparable across the industry and therefore within and between food safety jurisdictions.
- Lack of kits and available antibodies for ‘non-traditional’ species.
- Cost and time requirements; a high-throughput method is likely to provide an easier alternative for the industry.
- Limited access to pure, well-characterized growth factor standards hampers validation.

Risk assessment

- No thresholds or conventional measurements for ‘safe’ growth factor levels makes interpretation challenging and requires an understanding of suitable LODs.

## 4. Discussion

The CMSI multi-stakeholder format proved valuable insight into aligning technical work with regulatory expectations. By convening experts from academia, industry, government, and non-profits, the initiative enabled open discussion of challenges that no single actor could address alone. Participants consistently highlighted that progress in medium safety assessment will require pre-competitive collaboration. Multi-disciplinary consortia, involving analytical laboratories, researchers, suppliers, producers, and regulators, will be essential for method validation and data harmonisation. Collaborative validation and shared infrastructure were emphasised as necessary to overcome both technical and economic barriers, particularly the high cost of method development, limited access to reference standards, and a lack of publicly available datasets.

### 4.1. Integrating Workshop Priorities with Case Study Findings

The ELISA development work underscores the practical challenges behind one of the workshop’s core priorities: residue detection. Specific issues such as matrix interference, inadequate sensitivity, and the lack of validated standards are not unique to growth factor detection, but mirror concerns raised across other workshop focus areas. For example:

- **Residue measurement methods:** ELISA results showed that growth factor detection can vary significantly depending on matrix effects and assay kit quality, echoing participants’ calls for interlaboratory validation and comparative evaluation with MS.
- **Certificates of Analysis (CoAs):** The lack of consistent reference materials and quality specifications parallels industry uncertainty around what safety-relevant attributes should be included in CoAs for medium inputs.
- **Metabolites and breakdown products:** The observation that growth factors degrade substantially during cooking, often to below detection limits, reflects the importance of systematically considering processing and post-harvest changes in any safety evaluation.
- **Open databases:** Workshop participants stressed the need for centralised repositories for growth factor and antibody performance data. The ELISA case study highlights how duplication of effort and inconsistent kit performance could be reduced through shared datasets.

Together, these findings highlight that residue detection is both a scientific and regulatory challenge, one that cannot be solved in isolation but must be integrated into a broader, harmonised safety framework.

### 4.2. Role of Cross-Sector Collaboration

The CM sector is characterised by diverse stakeholders, such as startups, ingredient suppliers, contract labs, regulators, and standards organisations. Each have partial but incomplete visibility over safety considerations. For example, workshop participants noted that suppliers often do not know what CoAs to develop for CM-specific ingredients; companies lack clarity on which tests regulators expect; and regulators lack access to validated datasets for decision-making.

Cross-sector collaboration offers solutions to these fragmented efforts by enabling shared priorities, resource pooling, and pre-competitive method development. For example, analytical method development could be pursued through consortia pooling resources to conduct interlaboratory validation of ELISA and MS protocols, reducing duplication and improving reproducibility across the sector.

Within this collaborative context, the Safety-Assessed Media Ingredient (SAMI) List has been developed within the CMSI to support risk-based assessment of culture medium ingredients by both categorising ingredients and developing screening-level benchmarks for safe use [6]. Current work is evaluating components that may require additional testing as they lack a long history of safe use, such as growth factors and other recombinant proteins, as well as complex mixtures such as hydrolysates. The SAMI framework offers a foundation for safety assessment, but standardised analytical methods and reference datasets are still needed to support residue detection and application of the framework. Similar to this project on growth factors, the SAMI List and categorisation framework were vetted by a diversity of stakeholders from the CM community.

Shared infrastructure, such as centralised databases, could allow companies to contribute anonymised datasets on growth factor residue levels, metabolite profiles, or hydrolysate characterisation. Regulators emphasised that harmonised protocols and reference datasets would greatly improve the efficiency of dossier review. At the same time, collaborative development of CoA templates and assessment methods, drawing on precedents from standards from other industries, such as the biopharmaceutical industry, could clarify expectations for suppliers and manufacturers. However, participants noted that medium formulations are often undisclosed and intellectual property issues will need to be addressed.

By aligning these efforts, the CMSI and its partners can reduce duplication, lower costs, and accelerate both regulatory approval and consumer trust.

### 4.3. Proposed Roadmap

Building on both the ELISA case study and the workshop outcomes, a practical roadmap for advancing culture medium safety assessment includes:

- **Consortium formation:** Establish multi-stakeholder working groups to pursue research, share resources, and design collaborative projects to address research priorities to validate and harmonise methods.
- **Baseline data generation:** Collect reference datasets for relevant components in conventional meat and CM.
- **Method development and validation:** Develop protocols for safety assessment (including sample extraction, preparation, and analysis), and pursue interlaboratory validation studies to ensure reproducibility, with clear criteria for sensitivity and specificity. Create fit-for-purpose approaches, balancing rigour with practicality and cost.
- **Certificates of Analysis:** Develop guidance on safety-relevant specifications for culture medium inputs.
- **Centralised, open databases:** Develop repositories hosted by neutral organisations to share validated methods, performance characteristics, and anonymised data. Such platforms would help reduce duplication, guide regulatory expectations, and build trust. There will be a need to develop data inclusion criteria for these open databases to ensure they are fit for industry implementation.
- **Standards engagement:** Collaborate with international bodies (e.g., ISO, AOAC, Codex) to formalise new methods and align terminology, specifications, and validation criteria.
- **Application to regulatory pathways:** Ensure that methods and datasets developed are directly translatable into regulatory dossiers and provide regulators with harmonised tools for evaluating safety and reducing uncertainty and review timelines.

### 4.4. Next Steps

The present work represents an early but important step toward establishing harmonised approaches for culture medium safety. Progress to date has been achieved through voluntary collaboration, but sustained advancement will require a more structured approach. Key next steps include securing sustained funding to support pre-competitive research and consortium development; sharing of data; expanding participation to ensure international alignment; and undertaking formal interlaboratory method validation studies to develop standardised analytical protocols that can be applied consistently across the sector.

## 5. Conclusions

Validated and harmonised analytical methods for culture medium components are essential to CM safety assessment. The CMSI workshop and case study identified urgent research needs and proposed collaborative pathways to address them, from residue detection to open data infrastructure. Moving from discussion to implementation will require sustained, cross-sector investment and coordination, supported by governmental commitment to fund and prioritise regulatory science. Such an investment will be critical to enable transparent, science-based regulatory review and build public trust in CM products.

## Figures and Tables

**Table 1:** Multus medium serum replacer formulations for SMA and SMB concentrated stock, added to DMEM-F12 at 10% *v*/*v*.

Ingredient	SMA (mg/L)	SMB (mg/L)
Albumin	1300	6000
Insulin	30	30
Transferrin	60.8	60.8
Sodium Selenite	0.0067	0.0067
Vitronectin	0.1	0.1
Fetuin	400	400
CD Lipid concentrate	0.01	0.01
Retinoic acid	0.3	0.3
L-ascorbic acid 2-phosphate	30	30
EGF	0.05	0.05
FGF2	0.0005	0.0005
TGFb	0.01125	0.025
IGF1	0.0375	0.1
PDGFbb	0.02	0.09
Lysate (Hypea 7404)	250	250
HGF	0.025	0.05
Folic Acid	37.5	50
Sodium Pyruvate	151.077	440
Cholesterol	0.45	0.45
Tocopherol	0.02	0.02

## Data Availability

The original contributions presented in this study are included in the article and Supplementary Materials. Further inquiries can be directed to the corresponding author.

## Supplementary Materials

The following supporting information can be downloaded at: https://www.mdpi.com/article/10.3390/foods15040783/s1, Supplementary Materials S1: Workshop Agenda; Table S1: ELISA plate sources and ranges for each growth factor target and species; Table S2: Concentrations of targets in non-heated FBS media, SMA and SMA media and Lamb meat, as determined by human, bovine and ovine ELISAs.
