# Supplementary material for: Standardising Culture Medium Safety Testing for Cultivated Meat: Outputs from a Workshop and Case Study
Source: Foods. 2026 Feb 21;15(4):783. doi: 10.3390/foods15040783 (PMC12939730; doi:10.3390/foods15040783)
Supplement: Supplementary file 1 [file foods-15-00783-s001.zip › Supplementary Materials S1 - CMSI Workshop Agenda.pdf]

Cultured Meat Safety Initiative: UK Media Safety Workshop

Developing standards for 'food-safe' culture media in cultivated meat and seafood applications

Wednesday, June 25, 2025, 9 AM - 4 PM
I-X, Translation and Innovation Hub (I-HUB) 5th Floor,
Imperial White City Campus, 84 Wood Lane, London W12 0BZ

Agenda

Breakfast/Check-in	8:30 - 9:00 am
Welcome & CMSI Overview (Cai Linton, Multus)	9:00 - 9:10 am
Participant Introductions	9:10 - 9:25 am
Presentations	
Context for Workshop (Jo Anne Shatkin, Vireo)	9:25 - 9:40 am
Media Component Categorization/Assessment (Kim Ong, Vireo)	9:40 - 10:00 am
Analytical Challenges - ELISA + LC MS/MS (Ruth Wonfor, Aberystwyth)	10:00 - 10:30 am
Coffee Break	10:30 - 10:50 am
Panel discussion: Media Supplier Responsibilities<br>Catherine Elton (Qkine), Jon Dempsey (Dempsey Consulting/Roslin), Martin Carballo Pacheco (Hoxton)	10:50 - 11:20 am
Presentation: How Standards are Developed (Max Ryadnov, NPL)	11:20 - 11:40 am
2-Minute Project Pitches & Open Discussion	11:40 - 12:10 pm
Group Discussion & Topic Voting	12:10 - 12:30 pm
Lunch	12:30 - 1:30 pm
Intro to Breakout Group Topics	1:30 - 1:40 pm
Breakout Session 1	1:40 - 2:30 pm

Goal: Participants will choose one of the topic areas to dive deep into
Example discussion questions:

- What research project(s) are needed to address this topic?
- What methods or approaches can be used?
- What are the challenges/needs of working on these projects?
- Who can lead or contribute to these projects?

Coffee Break	2:30 - 2:40 pm
Breakout Session 2	2:40 - 3:10 pm

Goal: Complete Breakout 1 discussion and develop a 5-sentence project description

Group Discussion: Synthesis & Takeaways	3:10 - 3:40 pm
Next Steps & Closing Remarks	3:40 - 4:00 pm

Pre-workshop Resources:

The workshop will begin with a brief overview of the Cultured Meat Safety Initiative and presentations on key topics in culture media safety. However, it is important that we hit the ground running, and we ask that participants familiarize themselves with the topics in advance.

Starting project ideas and research topics:

During the workshop, participants will identify standardization needs for 'food-safe' culture media they believe are the most urgent/important and develop action plans to tackle them. The session will begin with a list of potential research topics identified from previous work by Multus in collaboration with Aberystwyth University and Vireo Advisors, funded by the UK government, aiming to develop standardised methods for measuring residual growth factors in cultivated meat and seafood.

Please review this list and familiarize yourself with any topics you are interested in working on. Participants will have an opportunity to do a two-minute pitch and propose additional topics in the morning of the workshop.

- Starting research topics list

Relevant publications and reports:

We highly encourage participants to review these resources prior to the session to get up to speed on culture media safety:

- Background document on categorization scheme for media components
- EFSA Scientific Colloquium report on cell culture-derived foods and food ingredients
- BSI Strawman Standards Scope
- Report identifying common media components, summarizing current approaches to assessing culture media, and challenges
- Summary of UKRI project with Multus, Aberystwyth University, and partners
- List of potential Category 3 & 4 substances, safety concerns or questions and endpoints to consider
- Cell-cultured Meat Safety Infographic and Q&A (*in preparation)*

Resources can be found here. Additional resources will be added in advance of the workshop.

Participation Guidelines:

The working session will be held under Chatham House rule. The information discussed is free to use, but neither the identity nor affiliation of the speaker, nor that of any other participant, may be revealed.

There will be no audio/video recordings of the working session without permission. Information shared may be documented in written notes (without attribution unless requested by the speaker).

Divergent thinking (ideation) and convergent thinking (honing in on a topic) will both be important at different stages of this working session. Please plan to stay on topic and work towards the goal of an action plan!

About Us:

This Cultured Meat Safety Initiative (CMSI) Phase 3 workshop is organised by Multus in collaboration with Aberystwyth University, and workshop organizers from the Bezos Centre at Imperial College, Austrian Centre of Industrial Biotechnology (acib), Good Food Institute (GFI), New Harvest/FEASTS, Hoxton Farms, Ivy Farm, Qkine, RSSL and Vireo Advisors. Generous in kind support has been provided for venue space by Imperial College London.

The CMSI was launched in 2020 by New Harvest and Vireo Advisors to address critical technical, methodological, and informational challenges related to the safety evaluation of cultured meat and seafood (CM) products. CMSI Phases 1 and 2 convened industry and governmental stakeholders to identify cultured meat safety research priorities (Ong *et al.* 2021, Ong *et al.* 2023). Building on this work, CMSI Phase 3 aims to convene diverse stakeholders to create the infrastructure for shared knowledge, methods, and data that address the priorities identified in Phase 1 and 2. Our long-term goal is to coordinate and launch research efforts to develop and validate analytical methods and generate publicly available datasets to support transparent food risk assessments and policy-making processes. Read more about the CMSI and ongoing projects here.
