# Supplementary material for: Standardising Culture Medium Safety Testing for Cultivated Meat: Outputs from a Workshop and Case Study
Source: Foods. 2026 Feb 21;15(4):783. doi: 10.3390/foods15040783 (PMC12939730; doi:10.3390/foods15040783)
Supplement: Supplementary file 1 [file foods-15-00783-s001.zip › Table S1.pdf]

Table S1: ELISA plate sources and ranges for each growth factor target and species.

Growth Factor		Human	Bovine	Ovine
**EGF**	Source	Invitrogen, KHG0061	ELK Biotechnology, ELK9094	MyBioSource, MBS1602115
	Range (pg/mL)	3.9 - 250	160 – 10000	50 - 1500
**FGF-2**	Source	Invitrogen, KHG0021	Invitrogen, EB2RB	L S Bio, LS-F41567
	Range (pg/mL)	15.6 - 1000	4.1 - 1000	6.25 - 400
**HGF**	Source	Invitrogen, BMS2069INST	ELK Biotechnology, ELK6687	MyBioSource, MBS743494
	Range (pg/mL)	63 - 4000	15.63 - 1000	1000 - 25000
**IGF-1**	Source	Invitrogen, EH250RB	Invitrogen, EB6RB	Cusabio, CSB-E13753SH
	Range (ng/mL)	0.123 - 30	1.229 - 120	15.6 - 1000
**PDGF**	Source	Invitrogen, BMS2071	ELK Biotechnology, ELK0727	MyBioSource, MBS1603119
	Range (pg/mL)	31.3 - 2000	6.88 - 3000	37.5 - 2400
**TGF β1**	Source	Invitrogen, BMS249-4	ELK Biotechnology, ELK5836	Cusabio, CSB-EL023446SH
	Range (ng/mL)	0.031 - 2	0.031 - 2	15.6 - 1000

Legend. Company details: **Invitrogen**, Thermo Fisher, Waltham, MA, USA; **ELK Biotechnology**, Sugar Land, TX, USA; **MyBioSource**, San Diego, CA, USA; **L S Bio**, Newark, CA, USA; **Cusabio**, Houston, TX, USA.
