# Supplementary material for: Standardising Culture Medium Safety Testing for Cultivated Meat: Outputs from a Workshop and Case Study
Source: Foods. 2026 Feb 21;15(4):783. doi: 10.3390/foods15040783 (PMC12939730; doi:10.3390/foods15040783)
Supplement: Supplementary file 1 [file foods-15-00783-s001.zip › Table S2.pdf]

Table S2: Concentrations of targets in non-heated FBS media, SMA and SMA media and Lamb meat, as determined by human, bovine and ovine ELISAs. Data are expressed as means of either duplicate technical replicates for control (non-target species) samples or means of batches for main (target species) samples. Main samples tested on a relevant target species ELISA are demonstrated via bold text. ND demonstrates samples that were not detected below the ELISA range. – demonstrates where samples were above the standard curve range and require further dilution to test again.

	**Human**	**Bovine**	**Ovine**
**EGF**			
FBS media (ng/mL)	ND	**ND**	0.46
SMA media (ng/mL)	**78.49**	168	2.63
SMB media (ng/mL)	**81.07**	174	2.75
Lamb meat (pg/mg)	0.02	0.001	**ND**
**FGF-2**			
FBS media (pg/mL)	39.1	**ND**	ND
SMA media (pg/mL)	**277.68**	ND	ND
SMB media (pg/mL)	**570.47**	ND	ND
Lamb meat (pg/mg)	8.5	0.48	**0.42**
**HGF**			
FBS media (pg/mL)	ND	**14.84**	2,203.5
SMA media (pg/mL)	**16,540.88**	150.65	ND
SMB media (pg/mL)	**21,347.8**	195.77	ND
Lamb meat (pg/mg)	ND	0.42	**6.97**
**IGF-1**			
FBS media (ng/mL)	ND	**1.53**	ND
SMA media (ng/mL)	**0.3**	7.2	31.08
SMB media (ng/mL)	**2.01**	3.32	24.93
Lamb meat (ng/mg)	0.003	0.12	**2.84**
**PDGF**			
FBS media	ND	**ND**	ND
SMA media (ng/mL)	**8.54**	2.13	10,582.07
SMB media (ng/mL)	**42.5**	1.59	-
Lamb meat (pg/mg)	0.77	4.31	**ND**
**TGF β1**			
FBS media (pg/mL)	38.5	**ND**	ND
SMA media (pg/mL)	**ND**	ND	ND
SMB media (pg/mL)	**ND**	ND	ND
Lamb meat (pg/mg)	0.23	0.96	**ND**
